# New ELISA Kits using C3 Binding Glycoprotein from *Cuscuta europea* Detect Mainly IgM CIC in Rheumatoid Arthritis and Progressive Systemic Sclerosis, but not in Systemic Lupus Erythematosus

**DOI:** 10.1080/10446670310001626544

**Published:** 2003

**Authors:** Spaska Angelova Stanilova, Emil Slavov Slavov

**Affiliations:** Department of Molecular Biology and Immunology Medical Faculty Thracian University Armeijska 11 St. Stara Zagora 6000 Bulgaria

## Abstract

Elevated levels of circulating immune complexes (CIC), containing IgG, IgM or IgA antibodies were detected in the sera of patients with autoimmune diseases. This might indicate a different biological meaning of the three isotypes of immunoglobulin (Ig) in the CIC. Each CIC assay detected only certain classes and subclasses of Ig in CIC material or fixed complement protein. In this study, a new method based on C3binding glycoprotein named CIF-ELISA and a well-known method ANTI-C3 ELISA, were used for quantitative assessment of IgM-CIC, IgG-CIC and IgA-CIC levels in human sera. A modified CIF-ELISA and ANTI-C3 ELISA for simultaneous detection of CIC, containing IgG, IgM and IgA, (stCIC), were also performed. The assays were evaluated on the same specially prepared samples: 55 normal sera, 99 sera from rheumatoid arthritis (RA), 88 sera from systemic lupus erythematosus (SLE), and 27 sera from progressive systemic sclerosis (PSS). We found that the sensitivity of the tests used varied depending on the diseases studied. CIF-ELISA displayed higher sensitivity of IgM-CIC when compared to ANTI-C3 ELISA in RA patients (40.0 and 20.95%, respectively) and PSS (44.43 and 37.04%, respectively). Results for the sensitivity of IgA-CIC were in adverse direction in the RA group (14.28 and 19.05%) and PSS (14.81 and 25.93%) by both methods. It was also established that the concordance of IgM-CIC positives by both methods was 48.84% in RA and 46.67% in PSS, while in SLE it was 18.78%. These results are most probably due to the different assay abilities to detect antibody isotype of the CIC material and help to explain what specific role each Ig isotype in CIC has in the course of the disease.


*Clinical & Developmental Immunology*, June–December 2003 Vol. 10 (2–4), pp. 111–117

Taylor & Francis healthsciences

### New ELISA Kits using C3 Binding Glycoprotein from *Cuscuta europea* Detect Mainly IgM CIC in Rheumatoid Arthritis and Progressive Systemic Sclerosis, but not in Systemic Lupus Erythematosus

SPASKA ANGELOVA STANILOVA and EMIL SLAVOV SLAVOV*

*Department of Molecular Biology and Immunology, Medical Faculty, Thracian University, Armeijska 11 St., 6000 Stara Zagora, Bulgaria*

Elevated levels of circulating immune complexes (CIC), containing IgG, IgM or IgA antibodies were detected in the sera of patients with autoimmune diseases. This might indicate a different biological meaning of the three isotypes of immunoglobulin (Ig) in the CIC. Each CIC assay detected only certain classes and subclasses of Ig in CIC material or fixed complement protein. In this study, a new method based on C3binding glycoprotein named CIF-ELISA and a well-known method ANTI-C3 ELISA, were used for quantitative assessment of IgM-CIC, IgG-CIC and IgA-CIC levels in human sera. A modified CIF-ELISA and ANTI-C3 ELISA for simultaneous detection of CIC, containing IgG, IgM and IgA, (stCIC), were also performed. The assays were evaluated on the same specially prepared samples: 55 normal sera, 99 sera from rheumatoid arthritis (RA), 88 sera from systemic lupus erythematosus (SLE), and 27 sera from progressive systemic sclerosis (PSS). We found that the sensitivity of the tests used varied depending on the diseases studied. CIF-ELISA displayed higher sensitivity of IgM-CIC when compared to ANTI-C3 ELISA in RA patients (40.0 and 20.95%, respectively) and PSS (44.43 and 37.04%, respectively). Results for the sensitivity of IgA-CIC were in adverse direction in the RA group (14.28 and 19.05%) and PSS (14.81 and 25.93%) by both methods. It was also established that the concordance of IgM-CIC positives by both methods was 48.84% in RA and 46.67% in PSS, while in SLE it was 18.78%. These results are most probably due to the different assay abilities to detect antibody isotype of the CIC material and help to explain what specific role each Ig isotype in CIC has in the course of the disease.

*Keywords*: ANTI-C3 ELISA; CIF-ELISA; C3bgp; Immune complex detection; Immune complex diseases

*Abbreviations*: AU, arbitrary unit; CIC, circulating immune complexes; CIF, C3 binding glycoprotein isolated from *Cuscuta europea* seeds; IgA-CIC, circulating immune complexes, containing IgA; IgM-CIC, circulating immune complexes, containing IgM; IC, immune complexes; PSS, progressive systemic sclerosis; RA, rheumatoid arthritis; SLE, systemic lupus erythematosus; stCIC, CIC levels, detected by the screening test

### INTRODUCTION

The importance of the immune complexes (IC) as a trigger of many autoimmune diseases is well established (Theofilopoulos and Dixon, 1980; Westedt *et al.*, 1986; Edwards and Cambridge, 1998). In the same time the dependency between the serum levels of the circulating immune complexes (CIC) and the disease activity is not yet completely elucidated (Ruddy and Moxley, 1994). One of the reasons for this problem may be related to the characteristics of the materials detected (McDougal *et al.*, 1982). It is well known that most of the tests used for quantitative assessment of CIC can detect preferentially IC, containing IgG (IgG-CIC). Therefore, the levels of CIC, containing other immunoglobulin isotypes (mainly IgM and IgA) will be undetected and the results obtained—uncompleted. On the other hand, the problem concerning the quantitative assessment of IgM-CIC and IgA-CIC is actual because of the currently established data for the elevated levels of these isotypes CIC in diseases as rheumatoid arthritis (RA), systemic lupus erythematosus (SLE), progressive systemic sclerosis (PSS), IgA nephropathy, vasculitis and other (Iida *et al.*, 1987; Miyazaki, 1990; Jarvis *et al.*, 1992; Jarvis *et al.*, 1995; Kashem *et al.*,

*Corresponding author. Tel.: +359-42-28-19-225. Fax: +359-42-600-705. E-mail: e_slav67@mf.uni-sz.bg

ISSN 1740-2522 print/ISSN 1740-2530 online © 2003 Taylor & Francis Ltd
DOI: 10.1080/10446670310001626544

1996; Coppo *et al.*, 1997; Blockmans and Bobbaers, 1998). All of the mentioned problems suggest that the usefulness of the tests for parallel detection of CIC, according different immunoglobulin isotypes contained will be more appropriate as a tool to better find out the diagnosis and prognosis of a diseases studied.

At present ANTI-C3 ELISA and the conglutinin test are the most used antigen-nonspecific assays able to detect CIC according to the immunoglobulin isotypes contained (IgG-CIC, IgM-CIC and IgA-CIC) (Iida *et al.*, 1987; Ruddy and Moxley, 1994; Lock and Unsworth, 2000). Unfortunately, both tests can detect false positive results (Aguado *et al.*, 1985; Holmskov *et al.*, 1992). It is obvious that a new sensitive and easy performing test for quantitative assessment of IgM-CIC and IgA-CIC would be actual. In our previous work, we reported developing of CIF-ELISA test for quantitative determination of IgG-CIC (Stanilova and Slavov, 2001). The following studies reveal that this test could be used for detection of IgM-CIC and IgA-CIC. In this study we have made and analyze quantitative assessment of the IgM-CIC, IgG-CIC and IgA-CIC results obtained by CIF-ELISA and ANTI-C3 ELISA in patients with RA, SLE and PSS.

### MATERIALS AND METHODS

### Collection and Storage of Samples

All samples were routinely processed within 4 h of collection. Blood samples were obtained from 214 patients of the three diseases: RA—99, SLE—88, and PSS—27 as well as 55 healthy adults volunteers for investigation. The patients were selected without accessory illnesses, according to the American College of Rheumatology criteria, as models of typical autoimmune diseases (Masi *et al.*, 1980; Tan *et al.*, 1982; Arnett *et al.*, 1988). The healthy HIV and HBsAg negative individuals were selected from Haemotransfusion center Stara Zagora blood donors. Serum samples from the patients and healthy adults were separated at room temperature (RT), and stored at −20°C until use.

### Assay System

#### *CIF-ELISA*

CIF-ELISA was performed according to the protocol, described previously with modifications (Stanilova and Slavov, 2001). Flat bottom polystyrene microtitter plates were coated with 20 μg/ml CIF in 0.2 M carbonate–bicarbonate buffer, pH 9.6 and incubated overnight at 4°C. After washing with 50 mM Tris–HCl, pH 7.2, containing 0.05% Tween 20 (2 × 5 min), the plates were blocked for 20 min with the same buffer. A measure of 100 μl of each tested serum, diluted 1:50 in sample buffer (0.1 M PBS, containing 1% BSA, 0.2%Tween 20, 1 mM $CaCl_2$ and $MgCl_2$) was added in duplicate and incubated for 60 min at RT. After three washes, 100 μl of the appropriate anti-human immunoglobulin antibodies peroxidase conjugate were added to each well as follow:

- Goat anti-human IgM (μ-chain specific) antibodies peroxidase conjugate diluted 1:15000 in washing buffer, for detection of IgM-CIC.
- Goat anti-human IgA (α-chain specific) antibodies peroxidase conjugate diluted 1:10000 in washing buffer, for detection of IgA-CIC.
- Goat anti-human IgG (γ-chain specific) antibodies peroxidase conjugate diluted 1:10000 in washing buffer, for detection of IgG-CIC.
- Polyvalent goat anti-human IgG,A,M (γ,α and μ-chain specific) antibodies peroxidase conjugate diluted 1:20000 in washing buffer, for simultaneous detection of IgG-CIC, IgM-CIC and IgA-CIC, named screening sCIF-ELISA.

All immunoconjugates were incubated for 60 min at RT. After the washing procedure, 100 μl of the substrate solution was added to each well and incubated for 10 min at RT. The developed colour reaction was stopped with 10% $H_2SO_4$, and OD units at 492 nm were measured on an ELISA plate reader (Rosys Anthos 2010, Austria).

#### *ANTI-C3 ELISA*

It was performed as described by Pereira *et al.* (1980) with slight modification. In brief, the microtiter plates were incubated overnight at 4°C with 10 μg/ml monospecific goat IgG antibodies against human C3 component of complement, diluted in carbonate–bicarbonate buffer, pH 9.6. After washing procedure (2 × 5 min) and blocking the unreacted sites (for 20 min), the wells were incubated with 100 μl of the tested sera diluted 1:50 in 0.1 M PBS, containing 1%BSA and 0.2% Tween 20 for 60 min at RT. Following 3 washes (3 × 5 min) goat anti-human IgM (μ-chain specific) conjugate diluted 1:15000 in washing buffer; or goat anti-human IgG (γ-chain specific) conjugate diluted 1:10000 in washing buffer; or goat anti-human IgA (α-chain specific) conjugate diluted 1:10000 in the same buffer; or polyvalent goat anti-human IgG,A,M (γ,α and μ-chain specific) conjugate diluted 1:20000 in washing buffer were added to each well. Each conjugate was incubated for 60 min at RT. The developed colour reaction was stopped with 10% $H_2SO_4$, and OD units at 492 nm were measured on an ELISA plate reader (Rosys Anthos 2010, Austria).

### Running the Samples

All sera were tested in parallel for quantitative assessment of IgG-CIC, IgM-CIC, IgA-CIC and stCIC, by both assays used. Each serum was tested in duplicate according to performance characteristics of the tests used, and the results obtained in OD units were calculated as percent of positive controls (arbitrary units—AU). The sera shown OD value higher than $\overline{X} \pm 2SD$ was used as positive controls.

TABLE I Mean value (*X*), standard deviation (SD) and range ($X \pm 2SD$) of IgM-CIC and IgA-CIC detected for control group of healthy blood samples by CIF-ELISA, ANTI-C3 ELISA, and for total CIC levels by screening CIF-ELISA, ANTI-C3 ELISA

| | IgM-CIC levels | | | IgA-CIC levels | | | CIC levels (by screening tests) | | |
|---|---|---|---|---|---|---|---|---|---|
| ASSAYS | $X$ | SD | $X \pm 2SD$ | $X$ | SD | $X \pm 2SD$ | $X$ | SD | $X \pm 2SD$ |
| CIF-ELISA | 51.15 | 18.71 | 13.73 ÷ 88.57 | 14.76 | 9.98 | 0 ÷ 34.72 | 51.70 | 14.09 | 23.52 ÷ 79.88 |
| ANTI-C3 ELISA | 32.38 | 15.54 | 1.3 ÷ 63.46 | 29.63 | 20.17 | 0 ÷ 69.97 | 67.09 | 10.69 | 45.71 ÷ 88.74 |

The results are expressed as percent of positive control—AU.

### Special Reagents and Purified Proteins

The C3 binding glycoprotein (CIF) was isolated from the *C. europea* seeds as described previously (Zhelev *et al.*, 1994). Sigma purchased goat anti-human C3 antibodies; goat anti-human IgM-peroxidase conjugate; goat anti-human IgG peroxidase conjugate; goat anti-human IgA-peroxidase conjugate, polyvalent goat-anti human IgG,A,M peroxidase conjugate and all reagents for buffers.

Statistical analysis was made by Student's criteria, Dispersion analysis (*F*-test), $\chi^2$-test and Spearman correlation analysis.

### RESULTS

### IgM-CIC Quantitative Assessment in Patients' and Healthy Blood Donors' Sera

The serum levels of IgM-CIC, detected in the group of the healthy adult volunteers were used to determine the normal values of this isotype CIC in the healthy population. The results obtained by CIF-ELISA and ANTI-C3 ELISA allows us to calculate the normal range $X \pm 2SD$ of IgM-CIC, which represented that over 95% of test results with normal sera were negative (specificity) for both methods used. The simultaneously tested panel of patients' sera with the control group was used to choose the suitable positive controls (higher CIC level sera) for each assay and to calculate AU as percent of positive control (Table I).

Test results obtained for the control and patient groups are shown in Fig. 1. It was established that the IgM-CIC levels, detected in the patient groups' were significantly higher than the control ones ($p < 0,05$) for both methods used. *F*-test, which tested whether the ratio of the two variances estimated was significantly greater, verified that there were significant differences between the data of the control and all patients groups obtained by each of the assays. The greatest *F*-value was obtained for CIF-ELISA compared to the ANTI-C3 ELISA in all disease groups studied. That trend was most evident for PSS group, where *F*-value were 50.09 for CIF-ELISA and $F = 25.11$ for ANTI-C3 ELISA. For the RA group estimated *F* were 47.87 for CIF-ELISA and $F = 7.72$ for ANTI-C3 ELISA. Only for SLE group the *F*-values were nearly equal ($F = 23.71$ and 22.46, respectively).

Correlation coefficient (*r*), between the results from the two of the assays revealed a well-expressed correlation

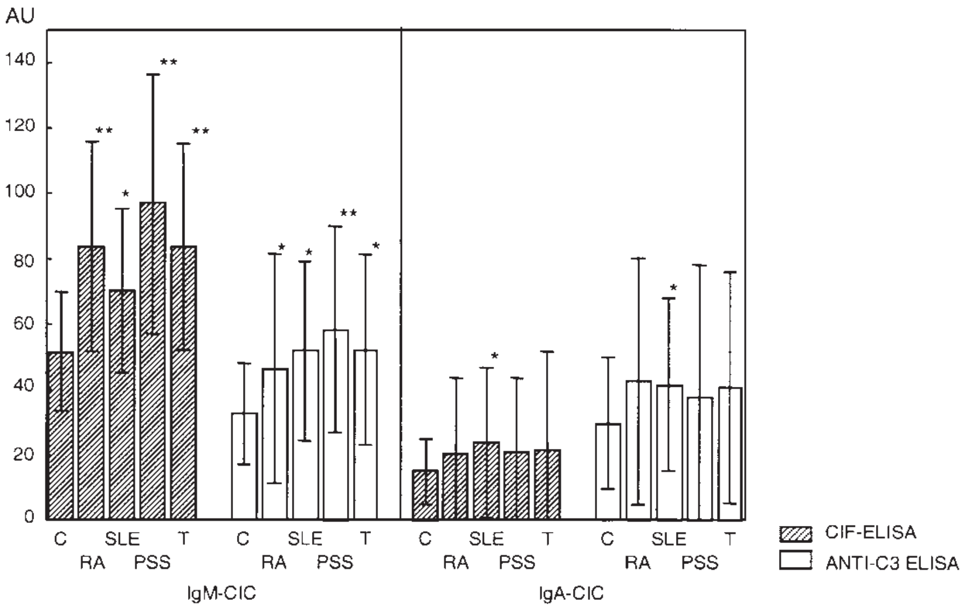

FIGURE 1 Mean and standard deviation values of IgM containing CIC and IgA containing CIC in control and patient groups detected by CIF-ELISA and ANTI-C3 ELISA. The data are presented in AU. With * and ** are presented statistically significant differences ($p < 0.05$, and $p < 0.01$, respectively) between control and patient groups. C- control group; T - total.

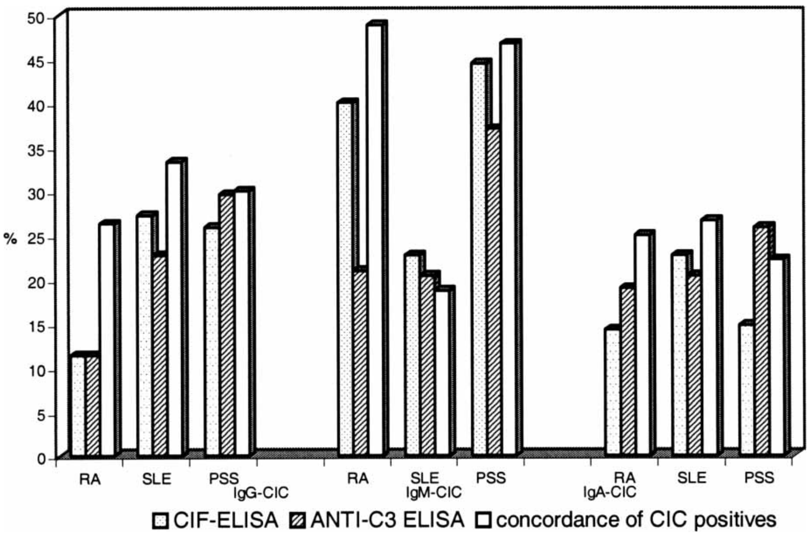

FIGURE 2 The Relative sensitivity and Concordance of IgG-CIC, IgM-CIC and IgA-CIC positive sera in patient groups detected by CIF-ELISA and ANTI-C3 ELISA. Relative sensitivity is calculated as percent (%) of positive sera. Concordance is calculated by the formula: the number of double positives as a percentage of the total number of single and double positive samples.

between the IgM-CIC levels, for RA ($r = 0.53, p < 0.01$) and PSS ($r = 0.50, p < 0.01$) groups. The same analysis of IgM-CIC data for SLE group revealed a slight, not statistically significant correlation ($r = 0.01$, $p > 0.05$).

The relative sensitivity of the assays used, was calculated as the number of patient sera with positive results divided to the number of patient sera tested and was presented as percent in Fig. 2. It was found that the relative sensitivity varied depending on the diseases studied. The relative sensitivity was higher by CIF-ELISA compared to ANTI-C3 ELISA for RA (40.0 and 20.95%, respectively) and PSS (44.43 and 37.04%) samples, whereas it was almost equal for SLE (22.73 and 20.45%).

For determining the degree of the coincidence between the two assays we calculated:

$$\%\text{Concordance} = \frac{100 \times \text{No. of anti}-\text{C3}^{+}/\text{CIF}^{+}}{(\text{No. of anti}-\text{C3}^{+} + \text{No. of CIF}^{+}) - \text{No. of anti}-\text{C3}^{+}/\text{CIF}^{+}}$$

(i.e. the number of double positives as a percentage of the total number of single and double positive samples).

This concordance of IgM-CIC positive sera was 48.84% for RA, 46.67% for PSS and 18.78% for SLE patients as shown in Fig. 2.

### Quantitative Assessment of IgA-CIC in Patients' and Healthy Blood Donors' Sera

The normal range of IgA-CIC levels was estimated upon the IgA-CIC levels detected from 55 healthy blood donor sera, and calculated as described for IgM-CIC (Table I).

The mean values of all patients' groups, detected by CIF-ELISA and ANTI-C3 ELISA were higher than the controls, however, statistically significant increased of IgA-CIC compared to the controls were detected for SLE group ($p < 0.01$, by both methods) alone, as shown in Fig. 1.

$F$-values calculated for ANTI-C3 ELISA in RA ($F = 5.49$) and SLE ($F = 8.0$) groups were higher than the $F$-values for CIF-ELISA in the same groups ($F = 2.38$ and $F = 7.31$, respectively). The $F$-values, detected for CIF-ELISA and ANTI-C3 ELISA in PSS group were nearly equal ($F = 2.37$ and $F = 1.38$, respectively), showing a statistically insignificant increase of IgA-CIC in this patients' group.

The correlation analysis established a well expressed correlation between CIF-ELISA and ANTI-C3 ELISA data of IgA-CIC levels for RA ($r = 0.34$, $p < 0.01$) and PSS ($r = 0.62$, $p < 0.01$) groups. This analysis also showed a weak, not significant correlation between the two tests for SLE group ($r = 0.07$, $p > 0.05$).

The relative sensitivity for IgA-CIC assessment was presented in Fig. 2. It was higher by ANTI-C3 ELISA compared to CIF-ELISA for RA (19.0 and 14.3%, respectively) and PSS (25.93 and 14.81%) samples, whereas it was almost equal for SLE (20.45 and 22.73%).

The concordance of IgA-CIC positive sera was 25.0% for RA group, 22.2% for PSS and 26.7% for SLE patient group as shown in Fig. 2.

### Quantitative Assessment of CIC in Patients' and Healthy Blood Donors' Sera by the Screening CIF-ELISA and ANTI-C3 ELISA

The CIC values of 55 healthy blood donor sera, detected by the screening CIF-ELISA (sCIF-ELISA) and screening ANTI-C3 ELISA (sANTI-C3 ELISA) were used to define

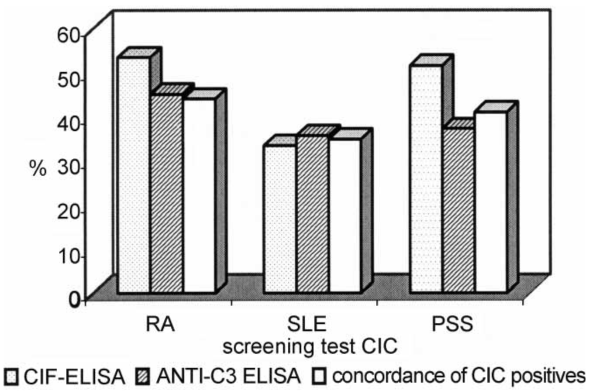

FIGURE 3 The Relative sensitivity and Concordance of total CIC (stCIC) positive sera in patient groups detected by CIF-ELISA and ANTI-C3 ELISA. The relative sensitivity and concordance are calculated as in Fig. 2.

the normal range of stCIC in the healthy population and the results are shown in Table I. The stCIC levels detected in patients' group were significantly higher than the control ones, by both methods ($p < 0.01$).

$F$-values detected by sCIF-ELISA were higher than those detected by sANTI-C3 ELISA. For the patients groups the results were as follow: for RA—$F = 50.79$ by sCIF-ELISA and $F = 29.28$ by sANTI-C3 ELISA, for SLE—$F = 28.84$ and $F = 6.97$ and for PSS—$F = 47.81$ and $F = 4.85$, respectively. A well-expressed correlation between sCIF-ELISA and sANTI-C3 ELISA stCIC levels for RA ($r = 0.35$, $p < 0.01$), SLE ($r = 0.36$, $p < 0.05$) and PSS ($r = 0.36$, $p < 0.05$) groups was observed.

The relative sensitivity of sCIF-ELISA and sANTI-C3 ELISA assessment of stCIC, are shown in Fig. 3. It was higher by CIF-ELISA for RA and PSS groups, while for SLE group the stCIC positives, detected by both methods are nearly equal. The concordance of stCIC positive sera was 43.9% for RA group, 40.97% for PSS group and 34.78% for SLE serum samples as shown in Fig. 3.

### Distribution of the CIC Positives Sera, Depends on the Immunoglobulin Isotype Contained

It was also investigated the percent of the distribution of IgM-CIC, IgA-CIC and IgG-CIC in the sera studied. The results obtained are presented in Table II. Both methods used demonstrate three different groups of positive sera. The first one was positive by a single isotype CIC, the second one was simultaneously positive sera by two isotypes CIC, followed by a third group with simultaneously positive sera by all three isotypes CIC. We established that the positive sera by one isotype CIC predominated over positive sera, contained two isotypes CIC simultaneously while triple positives (IgG, IgM and IgA-CIC) was the least part. When was used CIF-ELISA the highest percent of IgM-CIC positive sera were detected, while by ANTI-C3 ELISA the highest percent of sera were IgA-CIC positive.

TABLE II Distribution of the patients' sera with elevated CIC levels, detected by CIF-ELISA and ANTI-C3 ELISA according to the immunoglobulin isotype CIC or isotype CIC combinations contained

| Diagnosis | Total % of CIC positives | | IgG-CIC | | IgM-CIC | | IgA-CIC | | IgG+IgM-CIC | | IgG+IgA-CIC | | IgM + IgA-CIC | | IgG+IgM+IgA-CIC | |
|---|---|---|---|---|---|---|---|---|---|---|---|---|---|---|---|---|
| | CIF-ELISA | AC3-ELISA | CIF-ELISA | AC3-ELISA | CIF-ELISA | AC3-ELISA | CIF-ELISA | AC3-ELISA | CIF-ELISA | AC3-ELISA | CIF-ELISA | AC3-ELISA | CIF-ELISA | AC3-ELISA | CIF-ELISA | AC3-ELISA |
| RA | 50.5 | 29.5 | 2.86 | 1.90 | **31.4** | 4.76 | 2.86 | 4.76 | 1.90 | 3.81 | 4.76 | 1.90 | 4.76 | 8.57 | 1.90 | 3.81 |
| SLE | 41.0 | 33.0 | 7.95 | 6.82 | **5.68** | 0 | 2.27 | 2.27 | 4.55 | 5.68 | 7.95 | 3.41 | 5.68 | 7.95 | 6.82 | 6.82 |
| PSS | 55.6 | 44.4 | 11.1 | 0 | **22.2** | 3.70 | 0 | 7.41 | 7.41 | 7.41 | 0 | 0 | 7.41 | 3.79 | 7.41 | 22.2 |
| TOTAL | 47.3 | 32.7 | 5.9 | 3.64 | 20 | 2.73 | 2.3 | 4.09 | 3.64 | 5.00 | 5.45 | 2.27 | 5.45 | 7.73 | 4.55 | 7.27 |

The results are presented as percent of all tested sera.

### DISCUSSION

The quantitative assessment of CIC could be made according to the immunoglobulin isotypes or antigen contained by CIC (Theofilopoulos and Dixon, 1980; Nezlin *et al.*, 1998). In this regard the choice of method for quantitative assessment of CIC might be very important to better establish the diagnosis, as well as conduct a precise survey of the disease stage and the course of the treatment used (Nydegger and Svehag, 1984; Reeback *et al.*, 1985; Svendsen *et al.*, 1995; Ahmed *et al.*, 1999). In our previous work, we reported a new quantitative method named CIF-ELISA as an appropriate method for IgG-CIC detection (Stanilova and Slavov, 2001). The following experiments revealed the ability of CIF-ELISA to detect CIC, containing different immunoglobulin isotypes. In this study, IgM-CIC and IgA-CIC quantitative assessment results by CIF-ELISA were compared to those by well-known ANTI-C3 ELISA method.

The panel of healthy blood donor sera tested by CIF-ELISA was used to determine the normal range of IgM-CIC, IgA-CIC and stCIC level. We found that more than 95% of the healthy blood donor sera studied were with normal levels of these isotypes CIC. The same isotypes CIC quantity, detected in patients' groups by CIF-ELISA were significantly higher than the control. These results allowed us to conclude that CIF-ELISA is able to distinguish the human sera with elevated IgM-CIC, IgA-CIC and stCIC levels from the normal ones.

Results obtained strongly suggest that Immunoglobulin isotypes distribution included in CIC composition from each patients group was different, which influenced the assay sensitivity. It was also supported by the fact that the percent of positive sera for both assay used calculated as % concordance was varied depending of IgG or IgM or IgA-CIC detection. The higher sensitivity of CIF-ELISA compared to ANTI-C3 ELISA toward IgG-CIC and IgA-CIC levels was shown for patients with SLE alone. For this patients group the higher concordance between two assays was established as well regarding IgG and IgA-CIC.

The presence of elevated levels of IgA-CIC in the pathogenesis of many autoimmune diseases is well established. They are related with the outcome of the erosive arthritis and vasculitis (Westedt *et al.*, 1986; Roccatello *et al.*, 1993; Danning *et al.*, 1998). We detected elevation in IgA-CIC levels by CIF-ELISA and ANTI-C3 ELISA, with a bit higher sensitivity of the ANTI-C3 ELISA in RA and PSS patients.

The higher sensitivity of CIF-ELISA compared to ANTI-C3 ELISA toward IgM-CIC levels has been demonstrated for patients with RA and PSS, but not for SLE. These results revealed that the quantity of IgM-CIC in patient sera depends on the disease and it is higher in RA compared to SLE. It is possible that the detected IgM-CIC in RA patient represents complexes of modified IgG antibodies and rheumatoid factors of monomeric IgM isotype, directed against them. The role of this immune complex type is considered by some authors (Ng *et al.*, 1988; Jarvis *et al.*, 1992; Ligier *et al.*, 1998). Another reason for IgM-CIC persisting in patients sera might be some disturbances in class switching, leading to elevation in IgM antibody production (Fedyk *et al.*, 1994). Further study is needed to clarify the role of IgM-CIC in the pathogenesis of the autoimmune diseases, as well as the role of IgM-CIC as a marker of disease activity, for which CIF-ELISA methods could be useful. One possible explanation for the data obtained is the fact that monomeric IgM hampers the C3 complement component fixation to CIC, and these immune complexes remain undetected by ANTI-C3 ELISA (Balestrieri *et al.*, 1984). Our previously reported results demonstrated that CIF might binds to immune complex, regardless of C3 fixing (Stanilova *et al.*, 1999; Slavov *et al.*, 2000). We suppose some advantages of CIF-ELISA as a method for quantitative assessment of IgM-CIC in sera of patients, particularly for RA disease.

Our results confirm the indispensability of the quantitative assessment of IgM-CIC, IgG-CIC and IgA-CIC in the autoimmune disease patient's sera. When the results obtained for IgM-CIC, IgG-CIC and IgA-CIC were analyzed in parallel, it became clear that some sera contained simultaneously elevated levels of both isotypes CIC. This trend was evident by CIF-ELISA as well as by ANTI-C3 ELISA. We found sera with simultaneously elevated IgG-CIC, IgM-CIC and IgA-CIC, sera with elevated levels of two isotypes CIC in different combination - IgG-CIC and IgM-CIC; IgG-CIC and IgA-CIC or IgM-CIC and IgA – CIC, as well as sera with elevated only one isotype CIC (IgG-CIC, IgM-CIC or IgA-CIC).

More precise analysis of the results obtained show that the percent of sera with elevated levels of only one isotype CIC or of different combination of the three isotypes CIC studied vary depending on the diagnosis. We found that in RA and PSS groups mainly persists IgM-CIC, while in SLE prevails IgG and IgA-CIC.

The data obtained suggest that only the parallel detection of the three main isotypes CIC could present a complete information about CIC quantity persisting in the serum. Unfortunately, at present the only way to detect these three isotypes CIC is to run in parallel three ELISA tests, detecting separately IgM-CIC, IgA-CIC and IgG-CIC. This diagnostic procedure is difficult to be performed, and its possibilities are limited. In this regard the development of a screening test, detecting simultaneously IgM-CIC, IgA-CIC and IgG-CIC is more valuable. In the existing literature we did not found data about the ELISA tests for screening detection of CIC, regardless of antibody isotypes. We developed modified ANTI-C3 ELISA and CIF-ELISA screening tests, using the polyvalent IgG, IgM and IgA immunoconjugate as a detecting antibody. Our results demonstrated a well-expressed capacity of screening tests for CIC. That detection is a simple and quick test for the diagnostic practice. The higher % of CIC positives detected by sCIF-ELISA compared to sANTI-C3 ELISA as well as the higher level of CIC positives simultaneously by both assays used, allowed us to conclude that sCIF-ELISA is more appropriate than sANTI-C3 ELISA for quantitative assessment of total stCIC level.

The ability of CIF-ELISA to detect IgM-CIC, IgA-CIC and IgG-CIC as well as to detect the total CIC level might transform it in an useful tool for the routine laboratory use.

### Acknowledgements

We wish to thanks to Prof. Yehuda Shoenfeld for his invaluable help and advice.

### References

Aguado, M.T., Lambris, J.D., Tsokos, G.C., Burger, R., Bitter-Suerman, D., Tamerius, J.D., Dixon, F.J. and Theofilopoulos, A.N. (1985) "Monoclonal antibodis against complement 3 neoantigens for detection of immune complexes and complement activation. Relationship between immune complex levels, state of C3 and numbers of receptors for C3b", *J. Clin. Invest.* **76**, 1418–1426.

Ahmed, E., Nityanand, S., Mustafa, A., Brismar, K. and Lefvert, A.K. (1999) "Anti-cardiolipin antibodies and circulating immune complexes in type 1 diabetes mellitus: increased prevalence and relation to vascular complication", *Clin. Exp. Immunol.* **115**, 255–259.

Arnett, F.C., Edworthy, S.M., Bloch, D.A., McShane, D.J., Fries, J.F., Cooper, N.S., Healey, L.A., Kaplan, S.R., Liang, M.H. and Luthra, H.S. (1988) "The American Rheumatism Association 1987 revised criteria for the classification of rheumatoid arthritis", *Arthritis Rheum.* **31**, 315–324.

Balestrieri, G., Tincani, A., Migliorini, P., Ferri, C., Cattaneo, R. and Bombardieri, S. (1984) "Inhibitory effect of IgM rheumatoid factor on immune complex solubilization capacity and inhibition of immune precipitation", *Arthritis Rheum.* **27**, 1130–1136.

Blockmans, D. and Bobbaers, H. (1998) "Inflammation phenomena in vasculitis: from immune complexes to less immune forms", *Acta Clin. Belg.* **53**, 83–91.

Coppo, R., Cirina, P., Amore, A., Sinico, R.A., Radice, A. and Rollino, C. (1997) "Properties of circulating IgA molecules in Henoch-Schönlein purpura nephritis with focus on neutrophil cytoplasmic antigen IgA binding (IgA-ANCA): new insight into a debated issue", *Nephrol. Dial. Transplant.* **12**, 2269–2276.

Danning, C.L., Illei, G.G. and Boumpas, D.T. (1998) "Vasculitis assosiated with primary rheumatologic diseases", *Curr. Opin. Rheumatol.* **10**, 58–65.

Edwards, J.C.W. and Cambridge, G. (1998) "Rheumatoid arthritis: the predictable effect of small immune complexes in which antibody is also antigen", *Br. J. Rheumatol.* **37**, 126–130.

Fedyk, E.R., Borrello, M.A., Brown, D.M. and Phipps, R.P. (1994) "Regulation of B cell tolerance and triggering by immune complexes", *Chem. Immunol.* **58**, 67–91.

Holmskov, U., Haas, H., Teisner, B., Andersen, O. and Jensenius, J.C. (1992) "Calcium- dependent and calcium-independent signals in the conglutinin-binding assay (KgBa) for immune complexes. Influence of anti-collagen-antibodies", *J. Immunol. Methods* **148**, 225–232.

Iida, K., Mitomo, K., Fujita, T. and Tamura, N. (1987) "A solid-phase anti-C3 assay for detection of immune complexes in six distinguished forms", *J. Immunol. Methods* **98**, 23–28.

Jarvis, J.N., Pousak, T. and Krenz, M. (1992) "Detection of IgM rheumatoid factor by enzyme-linked immunosorbent assay in children with juvenile rheumatoid arthritis: correlation with articular disease and laboratory abnormalities", *Pediatrics* **90**, 945–949.

Jarvis, J.N., Diebold, M.M., Chadwell, M.K., Iobidze, M. and Moore, H.T. (1995) "Composition and biological behaviour of immune complexes isolated from synovial fluid of patients with juvenile rheumatoid arthritis (JRA)", *Clin. Exp. Immunol.* **100**, 514–518.

Kashem, A., Endoh, M., Nomoto, Y., Sakai, H. and Nakazawa, H. (1996) "Monocyte superoxide generation and its IgA-receptor in IgA nephropathy", *Clin. Nephrol.* **45**, 1–9.

Ligier, S., Fortin, P.R. and Newkirk, M.M. (1998) "A new antibody in rheumatoid arthritis targeting glycated IgG: IgM anti-IgG-AGE", *Br. J. Rheumatol.* **37**, 1307–1314.

Lock, R.J. and Unsworth, D.J. (2000) "Measurement of immune complexes is not useful in routine clinical practice", *Ann. Clin. Biochem.* **37**, 253–261.

Masi, A.T., Rodnan, G.P., Medsger, T.A., Altman, R.D., D'Angelo, W.A., Fries, J.F., LeRoy, E.C., Kirsner, A.B., MacKenzie, A.H., McShane, D.J., Myers, A.R. and Sharp, G.C. (1980) "Preliminary criteria for the classification of systemic sclerosis (scleroderma)", *Arthritis Rheum.* **23**, 581–590.

McDougal, J.S., Hubbard, M., Strobel, P.L. and McDuffie, F.C. (1982) "Comparison of five assays for immune complexes in the rheumatic diseases. Performance characteristics of the assays", *J. Lab. Clin. Med.* **100**, 705–719.

Miyazaki, M. (1990) "Immunological abnormalities in family members of patients with IgA nephropathy", *Jpn. J. Med.* **29**, 469–477.

Nezlin, R., Alarcon-Segovia, D. and Shoenfeld, Y. (1998) "Immunochemical determination of DNA in immune complexes present in the circulation of patients with systemic lupus erythematosus", *J. Autoimmun.* **11**, 489–493.

Ng, Y.C., Peters, D.K. and Walport, M.J. (1988) "Monoclonal rheumatoid factor-IgG immune complexes", *Arthritis Rheum.* **31**, 99–107.

Nydegger, U.E. and Svehag, S.E. (1984) "Improved standardization in the quantitative estimation of soluble immune complexes making use of an international reference preparation. Results of a collaborative multicentre study", *Clin. Exp. Immunol.* **58**, 502–509.

Pereira, A.B., Theofilopoulos, A.N. and Dixon, F.J. (1980) "Detection and partial characterization of circulating immune complexes with solid- phase anti-C3", *J. Immunol.* **125**, 763–770.

Reeback, J.S., Silman, A.J., Holborow, E.J., Maini, R.N. and Hay, F.C. (1985) "Circulating immune complexes and rheumatoid arthritis: a comparison of different assay methods and their early predictive value for disease activity and outcome", *Ann. Rheum. Dis.* **44**, 79–82.

Roccatello, D., Picciotto, G., Torchio, M., Ropolo, R., Ferro, M., Franceschini, R., Quattrocchio, G., Cacace, G., Coppo, R., Sena, L.M., de Filippi, P.G. and Piccoli, G. (1993) "Removal systems of immunoglobulin A and immunoglobulin A containing complexes in IgA nephropathy and cirrhosis patients. The role of asialoglycoprotein receptors", *Lab. Invest.* **69**, 714–723.

Ruddy, S. and Moxley, G. (1994) "Clinical utility of assays for immune complexes and complement", *Diagn. Lab. Immunol.* **14**, 387–400.

Stanilova, S.A., Self, C.H. and Zhelev, Zh.D. (1999) "Studies on binding of complement inhibiting factor isolated from *Cuscuta europea* with immune complexes and their components", *Comptes rendus l'Academie bulgare Sciences* **52**, 105–108.

Stanilova, S.A. and Slavov, E.S. (2001) "Comparative study of circulating immune complexes quantity detection by three assays—CIF-ELISA, C1q-ELISA and anti-C3 ELISA", *J. Immunol. Methods* **253**, 13–21.

Slavov, E.S., Stanilova, S.A. and Zhelev, Zh.D. (2000) "Study on interaction between antigen–antibody immune complexes, C3 complement and C3 binding glycoprotein from *Cuscuta europea* with immune complexes and their components", *Comptes rendus l' Academie Bulgare Sciences* **53**, 81–84.

Svendsen, A., Holmskov, U., Petersen, P.H. and Jensenius, J.C. (1995) "Difference and radio plots: simple tools for improved presentation and interpretation of scientific data. Unexpected possibilities for the use of the conglutinin binding assay in inflammatory rheumatic diseases", *J. Immunol. Methods* **178**, 211–218.

Tan, E.M., Cohen, A.S., Fries, J.F., Masi, A.T., McShane, D.J., Rothfield, N.F., Schaller, J.G., Talal, N. and Winchester, R.J. (1982) "The 1982 revised criteria for the classification of systemic lupus erythematosus", *Arthritis Rheum.* **25**, 1–16.

Theofilopoulos, A.N. and Dixon, F.J. (1980) "Immune complexes in human diseases", *Am. J. Pathol.* **100**, 531–591.

Westedt, M.L., Daha, M.R., Baldwin, III, W.M., Stijnen, T. and Cats, A. (1986) "Serum immune complexes containing IgA appear to predict erosive arthritis in a longitudinal study in rheumatoid arthritis", *Ann. Rheum. Dis.* **45**, 809–815.

Zhelev, Zh.D., Stanilova, S.A. and Carpenter, B.G. (1994) "Isolation, partial characterization and complement inhibiting activity of a new glycoprotein from *Cuscuta europea*", *Biochem. Biophys. Res. Commun.* **202**, 186–194.

